# Catheter-Related Bloodstream Infection With Femoral Central Access Versus Internal Jugular Access in Patients Admitting to Medical Intensive Care Unit

**DOI:** 10.7759/cureus.29416

**Published:** 2022-09-21

**Authors:** Syed Bilal Hafeez, Arslan Ahmed, Aftab Akhtar, Wasib Ishtiaq, Najam Ul Sehar Javed, Kiran Abbas, Maryam Khan, Hammad Zafar, Areesha Jawed

**Affiliations:** 1 Department of Critical Care Medicine, Shifa International Hospital Islamabad, Islamabad, PAK; 2 Department of Internal Medicine, Shifa International Hospital Islamabad, Islamabad, PAK; 3 Internal Medicine, Aga Khan University Hospital, Karachi, PAK; 4 Department of Research, Shifa International Hospital Islamabad, Islamabad, PAK; 5 Department of Medicine, Jinnah Postgraduate Medical Centre, Karachi, PAK; 6 Department of Medicine, Dow University of Health Sciences, Civil Hospital Karachi, Karachi, PAK; 7 Department of Medicine, Services Hospital Lahore, Lahore, PAK

**Keywords:** internal jugular venous access, femoral access, colonization, crbsi, catheter related bloodstream infections, central venous access

## Abstract

Background

The current research focused on studying the pattern of catheter-related bloodstream infections (CRBSI) with femoral central access versus internal jugular access in patients admitted to the medical intensive care unit (ICU).

Methods

A cross-sectional study was conducted at the Department of Emergency Medicine, Shifa International Hospital, between March 4, 2022, and August 4, 2022. All individuals who presented to the ICU who needed a central venous catheter (CVC) for more than 48 hours were included. Catheter insertion was not permitted if the patient had a history of dermatitis or burns at the site of insertion or if the hemodialysis procedure necessitated the insertion of the catheter into a blood vessel. Three groups of patients were created: group A patients had been diagnosed with CRBSI; group B patients had catheter colonization (CC); and group C did not have CRBSI or CC. Standard microbiological methods were used to identify all of the bacteria collected from the cultures. All data was documented in a predefined pro forma.

Results

Overall, 20 (12.12%) patients had positive CRBSI, 68 (41.5%) had CC, and the remaining 46.3% of cultures were negative. Elderly populations were more prone to acquiring CRBSI showing a significant correlation between older age and CRBSI (p < 0.0001). CC was significantly associated with a longer duration of ICU stay, i.e., 30.3 ± 3.7 (p = 0.003). The absence of both CRBSI and CC was significantly associated with a lower duration of catheterization (11 ± 8.5 days in group C versus 22.1 ± 6.9 and 18.7 ± 7 days in groups A and B, respectively; p < 0.0001). Our study revealed a higher risk of CRBSI when the femoral access was compared to the internal jugular access (58.3% vs. 41.7%; p = 0.0008). The study did not find any significant association of CC with femoral or internal jugular access. Furthermore, a significantly higher rate of negative cultures was reported in patients with internal jugular access as compared to femoral vein access (85.8% vs. 14.2%; p = 0.007).

Conclusion

The need for routinely monitoring and observing the microbiological spectrum in patients receiving care in intensive care units is highlighted by the current investigation. The patients with internal jugular vein access had a decreased incidence of CRBSI and CC, while those with femoral access experienced CRBSI more frequently. *Escherichia coli* and *Pseudomonas aeruginosa* were the most frequently isolated germs, and both were resistant to various drugs that are used today. It is essential to regularly monitor the epidemiology of CRBSI in order to adopt preventative measures for infection prevention and control, such as staff education, strict hygiene standards, and a higher nurse-to-patient ratio.

## Introduction

A central venous catheter (CVC) is a widely used technique to deliver medicines that cannot be adequately administered orally or through a cannula in the forearm. It is also used in scenarios requiring intensive care, such as monitoring cardiac and venous status, as well as for haemodialysis necessitated for renal replacement therapy [1]. The process of placing a synthetic catheter includes inserting the catheter into either the subclavian vein or internal jugular vein or via the femoral vein in the inguinal region. The far-end tip is conventionally placed in the distal third of the superior vena cava or the inferior vena cava in the case of femoral access [2].

However, catheter-related bloodstream infections (CRBSI) are a rising concern. A retrospective study comprising patients older than 15 years old showed that the most common pathogen involved in central venous access-related bloodstream infection was coagulase-negative Staphylococcus [3]. CRBSI caused by bacteria accounts for 5-17% of the cases [4]. The clinical guidelines identify a case of CRSBI if the patient is febrile, has chills, and has other systemic sepsis manifestations of unknown origin [5]. The number of episodes of CRBSI occurring in the intensive care units (ICU) in the United States is around 16,000, with a mortality rate of 400-5000 patients every year. This also increases the hospitalization duration as well as the hospital bills incurred owing to the prolonged stay [3].

Marik et al. found no dissimilarity in the incidence of CRBSIs between the three locations, contradicting previous research that found a lower risk of such infections when the internal jugular was contrasted to the femoral site [6]. Two randomized controlled trials compared the risk of bloodstream infections caused by catheter placement at the femoral versus the subclavian/internal jugular sites and found no substantial dissimilarity. The attributable risk factors for bloodstream diseases due to catheter placement were similar in the femoral and subclavian locations. Veten et al., however, suggest that the internal jugular is the most preferred location (128/214, 60%) for placing CVCs in critically unwell children, with a reduced risk of infection being the most common rationale for this preference [7].

Whereas, Al-Sofyani and Uddin conclude that femoral lines are just as safe as internal jugular ones when it comes to CRBSI [8]. It further suggests that the prevalence of CRBSI cannot be reduced by avoiding femoral venous access. Catheter colonization (CC), CRBSI, and thrombosis are equally common in the jugular and femoral access sites in critically ill individuals admitted to the ICU.

Due to the scarcity of literature on the subject in our locality, we aimed to undertake the present investigation. The primary goal of the study was to assess the rates of CRBSI and CC in ICU patients with either a femoral vein catheter or an internal jugular catheter. The secondary goal of the study was to study the susceptibility pattern of the isolated microorganisms.

## Materials and methods

A cross-sectional study was conducted at the Department of Emergency Medicine, Shifa International Hospital, between March 4, 2022 and August 4, 2022. The study was initiated after receiving ethical approval from the Institutional Review Board (IRB) of Shifa International Hospitals Ltd. with reference number IRB/054-22.

A non-randomized consecutive technique for sampling was employed to recruit participants in the study. All individuals presented to the ICU who needed a CVC for more than 48 hours and had negative pre-catheter cultures were included. Patients with a history of dermatitis or burns at the site of insertion were excluded from the study. Furthermore, cases where CVC was inserted for hemodialysis procedures were also not included.

In this study, we followed the following strategy for the insertion and regulation of catheters: doctors used sterile-barrier procedures when inserting the catheters, including using full-body sterile drapes over the insertion site, washing their hands with surgical antiseptic, and donning all the required personal protective equipment. The insertion site was sterilized with 10% povidone-iodine. Silk suture was used to securely fasten the catheters to the skin after they were introduced percutaneously using the Seldinger method. After the line was placed, an occlusive dressing of dry sterile gauze was used for 24 hours before being replaced with a semipermeable sterile dressing. No antibacterial ointment or cream was applied to the areas of insertion. Hand hygiene precautions, such as washing with regular soap and water or using an alcohol-based hand wash, were taken before and after palpating catheter insertion sites and before accessing, repairing, or dressing catheters. As per hospital protocol, all admitted patients underwent pan cultures, including sputum cultures, blood cultures, and urine cultures on day zero and day seven. Blood cultures were also advised in case the patient developed any fever while he or she was in the ICU or if the patient developed septic shock.

All patients provided the following samples for culture: (1) after removing a CVC, doctors could examine the final 4.5 cm to see if the patient was colonized by bacteria; (2) two samples of blood, one taken directly from the catheter and the other from a peripheral vein. Lastly, in the event that blood could not be drawn from a peripheral vein, two blood samples were drawn through two different lumens of the catheter.

IDSA's published definitions of CC and CRBSI were utilized [9]. A semiquantitative culture of a catheter tip generated by the roll-plate method was regarded as successful if at least 15 colony-forming units (CFUs) were multiplied there, while quantitative samples required at least 1,000 CFUs to be considered successful.

If symptoms of bloodstream infection occur in a patient who has no known risk factors for such an infection, a diagnosis of CRBSI can be made if they meet either of the following criteria. (1) Colonization of a catheter and co-isolation of a pathogen in more than one peripheral blood culture. Two samples, one taken from the hub of a catheter and the other from a peripheral vein, require quantitative blood cultures that pass CRBSI's standards before they are considered positive. (2) Microorganisms cultured from blood collected at the catheter hub have a colony count at least three times higher than those cultured from blood collected at a peripheral vein. (3) The colony number for the patient's blood drawn from one catheter lumen is at least three times higher than the colony-forming units for the blood sample drawn through the other catheter lumen, according to the results of two quantitative blood cultures. However, the differential time to positive (DTP) is not something we utilize consistently. DTP is when bacteria multiply in a catheter hub blood sample at least two hours before they multiply in a peripheral vein blood sample.

Three groups of patients were created: group A patients had diagnosed CRBSI; group B patients had CC; and group C did not have CRBSI or CC. Standard microbiological methods were used to identify all of the bacteria collected from the cultures. Species-specific disk-agar diffusion assays for antibiotic susceptibility were conducted in accordance with the European Union Committee on Antimicrobial Susceptibility Testing (EUCAST) [10]. Tested antibiotics included ampicillin, ticarcillin, piperacillin ticarcillin clavulanate, cefazolin, cefotaxime ceftazidime, imipenem, ciprofloxacin, amikacin, tobramycin, gentamicin, colistin, tigecycline, and trimethoprim sulfamethoxazole. EUCAST standards were used to assess all susceptibility test findings.

All data were examined using the statistical package for social sciences (SPSS, IBM Corp., Armonk, NY). All categorical data including main reasons for ICU admissions, comorbidities, et cetera were listed as proportions. All continuous including length of stay, length of catheterization, etc., were presented as mean and standard deviation. The association of CRBSI with femoral and internal jugular access was determined using the Chi-square test, keeping the alpha <0.05 as the significance cut-off.

## Results

A total of 164 patients were included, with a total of 302 CVCs. The CVC insertion sites included the internal jugular 240 (79.5%) and the femoral vein 62 (20.5%). Overall, 20 (12.12%) patients had positive CRBSI (group A, with a total of 36 CVCs); 68 (41.5%) revealed CC (group B, with a total of 118 CVCs); and the remaining (46.3%) cultures were negative (no CRBSI or CC was detected; 148 CVCs).

Table 1 shows the sociodemographic and clinical parameters of the study population. Septic shock was the most common reason for ICU admission, with a mean length of stay and catheterization of 28.9 ± 5.2 days and 15.6 ± 6.7 days, respectively. The internal jugular was the most common site of catheterization, i.e., 240 (79.5%). Overall, the in-hospital mortality rate was 6.1%.

**Table 1:** Sociodemographic and clinical characteristics of study participants and central venous catheter ICU: intensive care unit; COPD: chronic obstructive pulmonary disease

Parameters	
Age (years)	52.1 ± 3.4
Main reason for ICU admission	
Septic shock	32 (19.5%)
Other etiologies of shock	19 (11.6%)
Community acquired pneumonia	24 (14.6%)
Exacerbations of COPD	30 (18.3%)
Coma	30 (18.3%)
Trauma	29 (17.7%)
Length of stay (days)	28.9 ± 5.2
Duration of catheterization (days)	15.6 ± 6.7
Comorbidities	
Diabetes mellitus	31 (18.9%)
Solid tumor	15 (9.1%)
Hematological malignancy	4 (2.4%)
Mechanical ventilation	69 (42.1%)
Sepsis at insertion	38 (23.2%)
One or more antibiotics before insertion	30 (18.3%)
Catheter site	
Internal jugular	240 (79.5%)
Femoral	62 (20.5%)
Parenteral Nutrition	51 (16.9%)
Insertion context	
Emergency	140 (46.4%)
Programmed	162 (53.6%)
In-hospital mortality	10 (6.1%)

Table 2 shows the post-stratification correlation of demographic and clinical parameters with different study groups (patients and CVC). Elderly populations were more prone to acquiring CRBSI, showing a significant correlation between older age and CRBSI (p < 0.0001). CC was significantly associated with a longer duration of ICU stay, i.e., 30.3 ± 3.7 (p = 0.003). The absence of both CRBSI and CC was significantly associated with a lower duration of catheterization (11 ± 8.5 days in group C versus 22.1 ± 6.9 and 18.7 ± 7 days in groups A and B, respectively; p < 0.0001).

**Table 2:** Association of clinical parameters with different study groups ICU: intensive care unit; COPD: chronic obstructive pulmonary disease

Parameters	Group A	Group B	Group C	P-value
Patients	20	68	76	
Age (years)	56.2 ± 3.91	51.9 ± 5.5	51.2 ± 3.1	<0.0001
Main reason for ICU admission				0.81
Septic shock	6 (30%)	15 (22.1%)	11 (14.5%)	
Other etiologies of shock	2 (10%)	9 (13.2%)	8 (10.5%)	
Community-acquired pneumonia	4 (20%)	9 (13.2%)	11 (14.5%)	
Exacerbations of COPD	3 (15%)	12 (17.6%)	15 (19.7%)	
Coma	2 (10%)	14 (20.6%)	14 (18.4%)	
Trauma	3 (15%)	9 (13.2%)	17 (22.4%)	
Length of stay (days)	28.9 ± 9.2	30.3 ± 3.7	27.8 ± 2.5	0.003
Duration of catheterization (days)	22.1 ± 6.9	18.7 ± 7	11 ± 8.5	<0.0001
Comorbidities				0.883
Diabetes mellitus	5 (25%)	16 (23.5%)	10 (13.2%)	
Solid tumor	3 (15%)	7 (10.3%)	5 (6.6%)	
Hematological malignancy	0 (0%)	2 (2.9%)	2 (2.6%)	
Mechanical ventilation	7 (35%)	29 (42.6%)	33 (43.4%)	0.788
Sepsis at insertion	9 (45%)	16 (23.5%)	13 (17.1%)	0.031
One or more antibiotics before insertion	7 (35%)	14 (20.6%)	9 (11.8%)	0.048
Mortality (patients)	2 (10%)	4 (5.9%)	4 (5.3%)	0.856
Central vein catheter	36	118	148	
Parenteral nutrition	5 (13.9%)	30 (25.4%)	16 (10.8%)	0.006
Insertion context				0.806
Emergency	15 (41.7%)	64 (54.2%)	61 (41.2%)	
Programmed	19 (52.8%)	68 (57.6%)	75 (50.7%)	

Our study revealed a higher risk of CRBSI when femoral access was compared to internal jugular access (58.3% vs. 41.7%; p = 0.0008). The study did not find any significant association of CC with femoral or internal jugular access. Furthermore, a significantly higher rate of negative cultures was reported in patients with internal jugular access as compared to femoral vein access (85.8% vs. 14.2%; p = 0.007) (Table 3).

**Table 3:** Association of CRBSI and CC with femoral venous catheters as compared to internal jugular venous catheters

Patient category	Internal jugular access	Femoral access	p-value
CRBSI (n=36)	21 (58.3%)	15 (41.7%)	0.0008
CC (n=118)	92 (78%)	26 (22%)	0.604
Neither CRBSI or CC (n=148)	127 (85.8%)	21 (14.2%)	0.007

Table 4 illustrates the distribution of susceptibility of *Escherichia coli* and *Pseudomonas aeruginosa* to different antibiotics utilized in common practice. 63.4% of the *E. coli* and 86.2% of *P. aeruginosa* were ampicillin resistant. Approximately half of the isolates were also resistant to ticarcillin and piperacillin.

**Table 4:** Rates of antimicrobial resistance and sensitivity among Gram-negative organisms most frequently isolated from the study population

Antimicrobial drug	*E. coli* (n=41)		*P. aeruginosa* (n=123)	
	Resistant	Sensitive	Resistant	Sensitive
Ampicillin	26 (63.4%)	15 (36.6%)	106 (86.2%)	17 (13.8%)
Ticarcillin	22 (53.7%)	19 (46.3%)	76 (61.8%)	47 (38.2%)
Piperacillin	21 (51.2%)	20 (48.8%)	84 (68.3%)	39 (31.7%)
Ticarcillin/clavulanic	19 (46.3%)	22 (53.7%)	69 (56.1%)	54 (43.9%)
Piperacillin/tazobactam	17 (41.5%)	24 (58.5%)	57 (46.3%)	66 (53.7%)
Cefazolin	19 (46.3%)	22 (53.7%)	113 (91.9%)	10 (8.1%)
Cefotaxime	10 (24.4%)	31 (75.6%)	110 (89.4%)	13 (10.6%)
Ceftazidime	8 (19.5%)	33 (80.5%)	67 (54.5%)	56 (45.5%)
Imipenem	5 (12.2%)	36 (87.8%)	44 (35.8%)	79 (64.2%)
Ciprofloxacin	9 (22%)	32 (78%)	39 (31.7%)	84 (68.3%)
Amikacin	8 (19.5%)	33 (80.5%)	30 (24.4%)	93 (75.6%)
Tobramycin	0 (0%)	41 (100%)	0 (0%)	123 (100%)
Gentamicin	13 (31.7%)	28 (68.3%)	93 (75.6%)	30 (24.4%)
Colistin	0 (0%)	41 (100%)	2 (1.6%)	121 (98.4%)
Tigecycline	0 (0%)	41 (100%)	0 (0%)	123 (100%)
TMP-SMX	18 (43.9%)	23 (56.1%)	97 (78.9%)	26 (21.1%)

## Discussion

The present study revealed a total CRBSI rate of 12.2% and a CC rate of 41.5%. We did not find any substantial difference between the femoral or internal jugular access and CC. In contrast to the present study, a study by Arvaniti et al. suggested that the risk of bacterial colonization was greater for the internal jugular as well as femoral catheters than for subclavian catheters, out of a total of 18,554 central vein catheters. This was found in both observational studies (9,331) and randomized controlled trials (5,482). Internal jugular and subclavian insertions were associated with a similar risk of CRBSI, while femoral insertions were associated with a higher risk (2.44 [95% CI, 1.25-4.75]), and internal jugular insertions were associated with a lower risk (0.55 [95% CI, 0.34-0.89]) [11].

A country's socioeconomic status and the type of intensive care unit being researched have a significant impact on the prevalence of CRBSI. Even though there are few exceptions, the incidence of CRBSI in industrialized countries is much lower than in developing or resource-scarce states [12].

A cohort study stated that with each episode of CRBSI, the length of hospital stay in patients with CRBSI increased by 19.6 days, with a corresponding cost of EUR 3124 [13]. Similarly, Pittet et al. [14] in their study highlighted that the CRBSI-associated odds ratio for mortality was up to 20.45 (95% CI, 18.9-22.1). Some studies also approximated around 500-4000 yearly deaths with an associated price of 3,124 EUR with every infection in Europe and $3,700-$29,000 in the United States [15,16]. It was also researched by Adler et al. that the hematological-oncology individuals' mortality rate due to CRBSI was 1.9% [17]. Research also suggests that the risk of CRBSI differs with the location of the central venous catheter. It has been proposed by Merrer et al. that an increased risk of CRBSI is seen with femoral access compared to other central venous access points [18]. The femoral veins are a reliable central venous access source, especially in urgent or emergency situations [18], despite the fact that femoral vein cannulation is often deemed less desirable due to greater complication rates. However, recent findings suggest that the magnitude of risk is not associated with the difference in insertion sites [5,19]. For example, Parienti et al. in their study identified no significant disparity in CRBSI rates between femoral venous and internal jugular access (0.93%, 3/324 vs 1.60%, 5/313) [20]. Shahar et al., over the course of three years, observed 496 cases of possible CRBSI, leading to 175 episodes in 119 patients. Within that time period, CRBSI affected 4.2% of patients while CC affected 4.8%. As was found, the diagnosis of CRBSI, with or without a peripheral culture, relies heavily on clinical evaluation and positive cultures [21].

As no study is free from limitations, we feel it is important to disclose some deficiencies in our study. First, the study used a non-probability technique to enroll the participants in the study; therefore, we cannot rule out the possibility that the findings we presented cannot be generalized and do not represent the entire population. Second, the sample size was small, and long-term follow-up was not maintained; most of the individuals were lost to follow-up. Thus, we recommend a larger and multicenter study be undertaken to minimize the flaws and strengthen the findings.

## Conclusions

The current study highlights the significance of regular monitoring and surveillance of the microbiological spectrum in patients who are catered in intensive care units. We found that CRBSI more frequently occurred in patients with femoral access, while patients with internal jugular vein access had a lower risk of CRBSI and CC. The most common organisms isolated were *E. coli* and *P. aeruginosa* and were resistant to several antibiotics commonly in use nowadays. In order to take proactive steps for infection control, inclusive of staff training, stringent cleanliness standards, and an appropriate nurse-to-patient ratio, it is crucial to continuously monitor the epidemiology of CRBSI.
